# Reply to Keen et al.: Dietz et al. modeling of climate tipping points is informative even if estimates are a probable lower bound

**DOI:** 10.1073/pnas.2201191119

**Published:** 2022-05-19

**Authors:** Simon Dietz, James Rising, Thomas Stoerk, Gernot Wagner

**Affiliations:** ^a^Department of Geography and Environment, London School of Economics and Political Science, London WC2A 2AE, United Kingdom;; ^b^Grantham Research Institute on Climate Change and the Environment, London School of Economics and Political Science, London WC2A 2AE, United Kingdom;; ^c^College of Earth, Ocean and Environment, University of Delaware, Newark, DE 19716;; ^d^Department of Environmental Studies, New York University, New York, NY 10003;; ^e^Robert F. Wagner Graduate School of Public Service, New York University, New York, NY 10003

Keen et al. (1) argue the conclusions and procedures in ref. 2 do not make sense, seemingly taking it as given that the economic impacts of climate tipping points will be larger than our estimates. We view the evidence base for the economic consequences of tipping points as highly uncertain but believe our paper can serve as an important foundation and tool for exploring these issues.

We agree our numbers are “probable underestimates” as we stated in our abstract. In ref. 2, *Discussion*, we explored why in detail. Reasons include missing climate tipping points, missing connections between tipping points, and missing impact channels. These have yet to be covered in the literature. Our aim was to integrate geophysically realistic models of climate tipping points with an economic model (e.g., refs. 3 and 4). Contrary to the impression given by Keen et al. (1), the literature we synthesized was developed in close association with the science. Nonetheless, clearly, the model can and should be extended, which we hope our methodology can help facilitate. We conducted extensive sensitivity analysis and reported several cost estimates much larger than our main specification (e.g., ref. 2, *SI Appendix*, sections 3.2.2, 3.2.5, and 3.2.7). We emphasized the consequent tail risk (see figure 2 of ref. 2).

Keen et al. (1) take issue with the results plotted in figure 5C of ref. 2, which shows the additional damages from climate tipping points as a function of global mean surface temperature. The estimates are obtained by running our model with and without climate tipping points and reporting the difference in consumption. Figure 5C of ref. 2 does not report the overall damages from climate change, which Keen et al. seem to imply. They are much larger. Possible misunderstandings aside, the damages from climate tipping points could certainly be larger than reported in figure 5C of ref. 2. We explained that a key uncertainty is whether climate change impacts the level of economic activity, or its growth rate. If it is the latter, both climate damages and the incremental damages from climate tipping points are much larger. With “pure” growth damages, the expected social cost of carbon is almost 100-fold higher than our main specification, and the contribution of climate tipping points is almost 3 times higher (ref. 2, *SI Appendix*, section 3.2.5). The latest evidence does not support pure growth damages (5), but the model admits this specification as a possibility.

The bulk of Keen et al.’s critique (1) focuses on nonmarket climate damages, a feature that is not even included in most of our analysis. We added a nonmarket damage function from ref. 6 to the model for the following sensitivity check: Suppose the economic impact of any given level of warming is larger, because of nonmarket impacts. Then, if climate tipping points increase warming even more, what is the extra cost of climate tipping points? Estimating nonmarket damages is notoriously difficult, which is why, conservatively, we do not include this function in most of our analysis. In any case, Keen et al.’s alternative calibration seems to assume that all US environmental controls in the 1990s were directed at climate change, which is incorrect.
